# Chrysodeixis chalcites nucleopolyhedrovirus (ChchNPV): Natural occurrence and efficacy as a biological insecticide on young banana plants in greenhouse and open-field conditions on the Canary Islands

**DOI:** 10.1371/journal.pone.0181384

**Published:** 2017-07-27

**Authors:** Ernesto Gabriel Fuentes, Estrella Hernández-Suárez, Oihane Simón, Trevor Williams, Primitivo Caballero

**Affiliations:** 1 Dpto. Protección Vegetal, Instituto Canario de Investigaciones Agrarias, Valle Guerra, La Laguna, Tenerife, Spain; 2 Instituto de Agrobiotecnología, CSIC-Gobierno de Navarra, Mutilva, Navarra, Spain; 3 Instituto de Ecología AC, Xalapa, Veracruz, Mexico; 4 Dpto. Producción Agraria, Universidad Pública de Navarra, Campus Arrosadía s/n, Pamplona, Navarra, Spain; Ecole des Mines d'Ales, FRANCE

## Abstract

*Chrysodeixis chalcites*, an important pest of banana crops on the Canary Islands, is usually controlled by chemical insecticides. The present study aimed to evaluate the efficacy of the most prevalent isolate of the Chrysodeixis chalcites nucleopolyhedrovirus (ChchNPV, *Baculoviridae*) as a biological insecticide. Overall the prevalence of ChchNPV infection in *C*. *chalcites* populations was 2.3% (103 infected larvae out of 4,438 sampled), but varied from 0–4.8% on Tenerife and was usually low (0–2%) on the other islands. On Tenerife, infected larvae were present at 11 out of 17 plantations sampled. The prevalence of infection in larvae on bananas grown under greenhouse structures was significantly higher (3%) than in open-field sites (1.4%). The ChchNPV-TF1 isolate was the most abundant and widespread of four genetic variants of the virus. Application of 1.0x10^9^ viral occlusion bodies (OBs)/l of ChchNPV-TF1 significantly reduced *C*. *chalcites* foliar damage in young banana plants as did commonly used pesticides, both in greenhouse and open-field sites. The insecticidal efficacy of ChchNPV-TF1 was similar to that of indoxacarb and a *Bacillus thuringiensis* (Bt)-based insecticide in one year of trials and similar to Bt in the following year of trails in greenhouse and field crops. However, larvae collected at different time intervals following virus treatments and reared in the laboratory experienced 2–7 fold more mortality than insects from conventional insecticide treatments. This suggests that the acquisition of lethal dose occurred over an extended period (up to 7 days) compared to a brief peak in larvae on plants treated with conventional insecticides. These results should prove useful for the registration of a ChchNPV-based insecticide for integrated management of this pest in banana crops on the Canary Islands.

## Introduction

Banana is the main crop on the Canary Island archipelago, covering an area of about 9,000 hectares and a total production of 381,983 tonnes in 2015 [1]. The tomato looper, *Chysodeixis chalcites* is a noctuid pest of bananas in the Canary Islands [2, 3]. Larvae often feed on banana fruit, causing damage to the epidermis that greatly reduces the market value of damaged fruit [3]. Control of this pest mainly involves the use of pesticides [2–4]. However, the low number of active compounds authorized for this crop in the European Union, and the repeated use of these products during the crop cycle [2, 5] favors the development of resistance [6], further reducing the effectiveness of the currently approved pesticides. In addition, since 2014 integrated pest management (IPM) is mandatory in crops grown in Spain (Royal decree 1311/2012, which incorporates Directive 2009/128/EEC). IPM is an ecologically based pest control strategy that favors natural mechanisms of pest control with minimal disruption from broad-spectrum pesticides [7, 8].

Lepidopteran nucleopolyhedroviruses (genus *Alphabaculovirus*, *Baculoviridae*) are virulent and selective pathogens with an established track-record as effective biological insecticides [9]. A single-nucleocapsid nucleopolyhedrovirus isolated from a single infected *C*. *chalcites* larva collected in banana crops in southern Tenerife, was characterized and named ChchNPV-TF1 [5]. Laboratory and small-scale greenhouse trials on young banana plants indicated that the high pathogenicity and fast speed of kill of this isolate favored its development as a biological insecticide for the control of this pest [5, 10]. Studies on other lepidopteran-nucleopolyhedrovirus pathosystems indicate that genetic diversity in the pathogen population has a marked influence on major phenotypic traits including pathogenicity, measured in terms of concentration-mortality metrics, speed of kill and production of progeny virus particles in each infected insect [11–13]. As these characteristics are of major importance to the efficacy of virus-based biological insecticides, the genetic identity of the virus is an issue of key importance when selecting the active material for production, formulation and field testing of virus-based products.

The objective of the present study was to estimate the genetic diversity of ChchNPV variants present in *C*. *chalcites* populations on the Canary Islands and to evaluate the insecticidal efficacy of ChchNPV-TF1, which was the most prevalent variant. Efficacy trials were performed in comparison with two frequently used insecticides in young banana plants grown in small-scale greenhouse and open-field conditions.

## Materials and methods

### Insects

The *C*. *chalcites* colony, used for artificial infestation of plants in greenhouse and open-field trials, was established with larvae collected in banana crops in southern Tenerife. This colony was maintained in the Instituto Canario de Investigaciones Agrarias (ICIA), Tenerife, at 25±1°C, 60–80% relative humidity and a photoperiod of 16:8 h (light:dark) on a semi-synthetic diet based on cornflour, wheatgerm and yeast [14]. Adults were fed *ad libitum* with 10% v/v honey solution.

### Prevalence and diversity of ChchNPV in *C*. *chalcites* populations in banana crops

During field surveys conducted in banana crops on the Canary Islands over a two-year period, from December 2012 to December 2014, plantations at 11 localities in Tenerife, 10 in La Palma, 7 in Gran Canaria, 4 in La Gomera and 2 in El Hierro were surveyed with the aim of determining the natural prevalence of ChchNPV infection (Table 1). No specific permissions were required for access to the plantations and the field studies did not involve endangered or protected species. Each location was classified as infested or not by *C*. *chalcites*, based on direct observation of feeding damage on leaves and fruit [E. Fuentes, pers. communication]. Thereafter in each infested plantation, larvae were collected from plants that showed *C*. *chalcites* feeding damage, both mature and immature plants. The sampling effort was similar for each location and involved a 4-hour period collecting larvae, or until ~100 larvae had been collected at each site. Collections were performed intermittently over the two year period. As the density of infestation varied, the number of larvae collected at each site varied accordingly (Table 1).

**Table 1:** ChchNPV surveys. Detailed information on ChchNPV surveys indicating the localities prospected, the crop system (OF: open-field and GH: mesh greenhouses), the number of surveys performed, the number of larvae collected and the larvae that died from virus infection and finally the ChchNPV isolates identified.

Canary Islands banana crops sample			Coordinates		Crop system	Surveys (n)	Larvae (n)	Isolates (n) (% prevalence)	ChchNPV isolates (number of larvae infected)
Island	Locality	Area	Lat. (N)	Long. (W)					
Tenerife	Fasnia	Fasnia	28° 13’ 45”	16° 24’ 57”	OF/GH	1	91	0 (0)	-
	Güimar	Puertito Güimar	28° 17’ 21”	16° 23’ 04”	GH	2	83	1 (1.2)	TF1 (1)
	Granadilla	San Isidro	28° 02’ 33”	16° 33’ 33”	GH	1	26	1 (3.9)	TF1 (1)
	Arona	El Cordero	28° 02’ 43”	16° 37’ 51”	GH	2	223	6 (2.7)	TF1 (5)
		El Fraile	28° 01’ 50”	16° 39’ 23”	GH	4	261	7 (2.7)	TF1 (6)
		Las Galletas	28° 01’ 13”	16° 40’ 24”	GH	2	102	1 (0.9)	TF1 (1)
		Valle Grande	28° 01’ 47”	16° 39’ 07”	GH	2	199	0 (0)	-
		Parque La Reina	28° 02’ 43”	16° 39’ 08”	GH	3	204	0 (0)	-
		Guaza	28° 01’ 35”	16° 39’ 11”	GH	1	34	1 (2.9)	TF3 (1)
	Adeje	Caldera del Rey	28° 04’ 19”	16° 43’ 02”	OF/GH	4	463	22 (4.8)	TF1 (12), TF2 (8)
	Guía Isora	Abama	28° 10’ 37”	16° 47’ 18”	OF	1	61	0 (0)	-
		Alcalá- Aurora	28° 13’ 08”	16° 49’ 40”	OF	1	81	0 (0)	-
	Buenavista	La Laja	28° 23’ 12”	16° 50’ 15”	GH	1	48	1 (2.1)	TF2 (1)
	Los Silos	Caleta	28° 23’ 07”	16° 49’ 39”	OF	1	56	2 (3.6)	TF1 (1), TF2 (1)
	Puerto Cruz	La Vera	28° 24’ 24”	16° 33’ 30”	OF	2	171	2 (1.2)	-
	Valle Guerra	Catesa	28° 31’ 20”	16° 24’ 43”	GH	3	367	12 (3.3)	TF1 (10)
	Tejina	Tejina Costa	28° 32’ 24”	16° 22’ 44”	GH	1	66	0 (0)	-
Total							2536	56(2.2)	TF1 (37), TF2 (10), TF3 (1)
La Palma	Fuencaliente	La Ballena	28° 29’ 11”	17° 52’ 14”	OF	3	167	3 (1.8)	TF1 (1)
	Los Llanos	El Remo	28° 33’ 18”	17° 53’ 15”	GH	3	128	1 (0.8)	TF1 (1)
		Charco verde	28° 34’ 26”	17° 53’ 55”	OF/GH	2	43	0 (0)	-
		Todoque	28° 36’ 50”	17° 54’ 20”	OF/GH	2	123	0 (0)	-
	Tijarafe	Tijarafe	28° 41’ 45”	17° 57’ 58”	GH	1	28	0 (0)	-
	Puntallana	Cabrera	28° 45’ 36”	17° 44’ 34”	OF	1	42	0 (0)	-
		Martín Luis	28° 42’ 59”	17° 44’ 26”	OF/GH	1	100	1 (1)	TF1 (1)
	Barlovento	Faro	28° 50’ 16”	17° 46’ 48”	OF	1	55	0 (0)	-
	San Andrés	Charco Azul	28° 48’ 25”	17° 45’ 47”	OF	1	21	0 (0)	-
	Mazo	El Pocito	28° 35’ 43”	17° 45’ 33”	OF	1	54	3 (5.6)	TF1 (3)
	Breña Alta	El Socorro	28° 39’ 34”	17° 46’ 32	OF	1	54	1 (1.9)	TF2 (1)
Total							815	9 (1.1)	TF1 (6), TF2 (1)
Gran Canaria	Vecindario	Pozo Izquierdo	27° 50’ 28”	15° 25’ 51”	GH	5	264	0 (0)	-
	Mogán	Arguineguín	27° 46’ 41”	15° 39’ 57”	OF	1	52	0 (0)	-
		Veneguera	27° 52’ 23”	15° 45’ 37”	OF	1	16	0 (0)	-
	La Aldea	La Aldea	27° 59’ 13”	15° 47’ 12”	OF/GH	1	36	0 (0)	-
	Gáldar	Gáldar Costa	28° 09’ 25”	15° 40’ 42”	OF/GH	1	72	0 (0)	-
		Gáldar	28° 08’ 11”	15° 38’ 36”	OF/GH	4	86	0 (0)	-
	Arucas	Bañaderos	28° 08’ 35”	15° 32’ 09”	OF	2	39	0 (0)	-
Total							565	0 (0)	-
La Gomera	San Sebastián	Chejelipes	28° 06’ 26”	17° 08’ 29	GH	1	38	0 (0)	-
	Hermigua	Hermigua	28° 10’ 37”	17° 10’ 59”	OF	1	21	0 (0)	-
	Vallehermoso	La Dama	28° 03’ 17”	17° 18’ 33”	GH	1	67	0 (0)	-
	ValleGranRey	Las Malezas	28° 05’ 33”	17° 20’ 13”	OF	1	63	1 (1.6)	TF3 (1)
Total							189	1 (0.5)	TF3 (1)
El Hierro	Frontera	El Matorral	27° 46’ 08”	18° 00’ 59”	GH	11	269	37 (13.8)	TF1 (33), TF3 (1), TF5 (3)
	El Pinar	Tacorón	27° 39’ 50”	18° 01’ 03”	GH	1	64	0	-
Total							333	37 (11.1)	TF1 (33), TF3 (1), TF5 (3)
Total overall							4,438	103 (2.3)	TF1 (76), TF2 (11), TF3 (3), TF5 (3)

Larvae were collected using a soft paintbrush, and individualized in 25 ml plastic pots containing diet. Larvae were maintained in a laboratory rearing chamber at 25 ± 2°C, 70 ± 15% RH and 16:8 hours (L:D) photoperiod, until death or pupation. Larvae were inspected daily and those died with the typical signs of polyhedrosis disease were individually frozen at -20°C for subsequent analysis. All larvae that died were observed using an optical microscope (x1000) to determine the presence of OBs.

To determine the identity of isolates from virus-killed field-collected insects, restriction endonuclease analysis (REN) was performed. For this, viral occlusion bodies (OBs) were purified from cadavers by homogenization in 0.1% (wt/vol) sodium dodecyl sulfate (SDS) and were filtered through muslin to remove debris. The resulting suspension was centrifuged at 3,800 x *g* for 5 minutes, OBs were resuspended in 500 μl of 0.1% SDS and centrifuged again at 3,800 x *g* for 5 minutes. OBs were finally resuspended in sterile distilled water. For viral DNA extraction, 100 μl of purified OB suspension was mixed with 100 μl of 0.5M Na_2_CO_3_, 50 μl of 10% SDS and distilled water to a final volume of 500 μl, incubated at 60°C for 10 minutes and centrifuged at 3,800 x *g* for 5 minutes. The resulting supernatant was treated with 25 μl of proteinase K (20 mg/ml) at 50°C for 30 minutes and then treated twice with 500 μl of TE-saturated phenol and once with 500 μl chloroform. The aqueous phase was recovered and DNA was precipitated by adding 1/10 volume 3M sodium acetate at pH 5.2 and 2.5 volumes of 100% cold ethanol. The DNA pellet was washed with 70% cold ethanol and resuspended in 50–100 μl 0.1x TE (Tris HCl-EDTA) buffer. For REN analysis, 2 μg of viral DNA were incubated with 5 units of *Bgl*II (Takara) at 37°C for 4 hours following the manufacturer's instructions. The reaction was stopped by adding 4 μl of loading buffer (0.25% w/v bromophenol blue, 40% w/v sucrose). Samples were loaded on 1% agarose gel and subjected to electrophoresis in TAE buffer (40 mM Tris-acetate, pH 8.0; 1 mM EDTA). The gel was stained using ethidium bromide, observed on a UV transilluminator and photographed using the GeneSnap Chemi-Doc package (BioRad, CA).

### Production of ChchNPV-TF1 OBs

For field trials, ChchNPV-TF1 isolate OBs were produced by inoculating fifth and sixth instar laboratory-reared *C*. *chalcites* larvae with 5.00x10^7^ OBs/ml or 9.02x10^8^ OBs/ml, respectively [5, 10]. OB inocula were suspended in 10% sucrose solution and 0.001% Fluorella blue food dye, and fed to larvae using the droplet feeding method [15]. Inoculated larvae were placed in 24 well tissue culture plates with semi-synthetic diet and incubated at 25°C. Larvae were checked daily for signs of polyhedrosis disease and dead insects were collected and stored at -20°C. OBs were collected by thawing infected insects, followed by homogenization, filtration through muslin and centrifugation at 3,800 x *g* for 5 minutes. The resulting pellet of OBs was resuspended in sterile water and OB concentration was determined by counting triplicate samples using an improved Neubauer hemocytometer (Superior Marienfield, Laude-Koeningshofen, Germany) under phase contrast microscopy at x400. Purified OBs were stored at 4°C until use. The identity of OBs produced for field trials was confirmed by REN analysis using *Bgl*II [5].

### Determining the optimal ChchNPV-TF1 concentration

To determine the most appropriate concentration for ChchNPV-TF1 OB application under field conditions we followed previous studies in which three viral concentrations were evaluated to estimate the most suitable concentration for use in field assays [10]. For this, trials were performed in 2013 using young banana plants (*Musa acuminata*, var. Dwarf Cavendish) of approximately 3 months old with 7 leaves and ~50 cm height. Plants were grown in 21 cm diameter pots in the experimental plots of the Instituto Canario de Investigaciones Agrarias (ICIA) (Tenerife, Spain). Trials were conducted in a plastic greenhouse of 600 m^2^ on the southern slope of Tenerife (28° 19' 2.8'' N; 16° 22' 59'' W) and in a 500 m^2^ open-field plot on the northern slope of the same island (28° 31' 43.5'' N; 16° 23' 13'' W).

Trials involved ChchNPV OBs applied at four different concentrations: 10^7^, 10^8^, 10^9^, 10^10^ OBs/l (equivalent to 10^10^, 10^11^, 10^12^ and 10^13^ OBs/ha assuming an application volume of 1000 l/ha), and water as the control. All treatments included 0.1% (v/v) Agral (Agro S.A., Madrid, Spain) wetter-sticker and were applied using a 2 l compressed-air hand sprayer (SOLO^®^ 402, Sindelfingen, Germany). The experimental design consisted of randomized plots with four replicates per treatment.

Experimental plots of 25 m^2^ comprised four rows of five plants each at a distance of 1 m from the adjacent plant (total = 20), of which 12 were border plants and 8 were central plants. Plants were artificially infested with *C*. *chalcites* eggs placed on the underside of the three youngest leaves of each plant. The number of eggs put in each plant varied from 50–150. Seven days later, when larvae had reached the second instar, plots were sprayed with a 1 l volume of each treatment (equivalent to 400 l/ha, which is usual for small banana plants). All applications were made between 8.00–11.00 am.

The efficacy of the different OB concentrations was calculated using the formula described by Henderson-Tilton [16]:

%Efficacy:1-(LtaxLcb/LcaxLtb)x100

Where Lta is the number of living larvae in plots after treatment, Ltb the number of living larvae in plots before treatment, Lca the number of living larvae in control plots after treatment and Lcb the number of living larvae in control plots before treatment.

In addition, the percentage of larval mortality at different time intervals after treatment was also determined. For this, 25 *C*. *chalcites* larvae were collected at random from the twelve border plants from each plot at time point 0 (immediately prior to the application of treatments), and at 1, 3, 5 and 7 days post-application. Larvae were reared individually in the laboratory in 25 ml plastic cups with semi-synthetic diet until death or pupation. Larvae that died with the characteristic signs of virus infection were stored at -20°C and REN analysis was performed subsequently to confirm that they had died due to ChchNPV-TF1 infection.

### Insecticidal efficacy of ChchNPV-TF1 in greenhouse and open-field trials

ChchNPV efficacy was compared with two conventional treatments in greenhouse and open-field trials during 2013 and 2015 at the same sites used for the concentration-mortality trials. Similarly, 3 month old banana plants var. Dwarf Cavendish, ~50 cm height and with 7 leaves grown in pots were used in greenhouse and open-field trials.

The experiments involved four treatments: (i) indoxacarb 30% WG (Steward, DuPont, Paris, France) applied at 0.04 g/l; (ii) *Bacillus thuringiensis* var. kurstaki 32% WG (Dipel DF, Kenogard, Barcelona, Spain) applied at 0.5 g/l; (iii) ChchNPV-TF1 OBs applied at 10^9^OBs/l and (iv) water control. All treatments included 0.1% (v/v) Agral wetter-sticker. Treatments were applied in a volume of 1 l using a 2 l capacity compressed-air hand sprayer (SOLO^®^ 402, Sindelfingen, Germany). All applications were made between 8.00 and 11.00 am.

In 2013, greenhouse and open-field experimental plots comprised four rows of 5 plants distributed over an area of 25 m^2^, as described for the concentration-mortality trials. In 2015, plots comprised 24 plants, distributed in 4 rows with 6 plants per row, with 16 border plants and 8 central plants. Plants were placed at 1 m intervals with a 1 m space between rows within each plot. Plots were separated by a 2 m high cloth curtain to avoid cross-contamination between treatments. In the greenhouse trial the pots were placed on the ground whereas in open-field pots were buried in the ground to avoid being blown over by the wind. In both years, greenhouse trials involved a fully randomized plot design with three replicates per treatment, while open-field trials were based on a Latin square design with four replicates per treatment.

Plants were artificially infested with eggs batches 7 days prior to treatments that were applied as described in the concentration-mortality trials. Similarly, insecticidal efficacy at 7 days post-application, and larval mortality in insects collected at 1, 3, 5 and 7 days post-application and reared in the laboratory until death or pupation, were measured as described in the concentration-mortality trials.

Foliar damage was also estimated. In 2013, damage was estimated by calculating the percentage increase in foliar damage in all the leaves (at least 7) of the eight central plants by counting the initial number of foliar perforations characteristics of *C*. *chalcites* feeding damage (old damage) on each leaf in each treatment prior to insecticidal treatments, and the final number of perforations, using the formula:

Damageincrease(%)=(Da–Db/Da)x100

Where Db is the damage before treatment, and Da the damage after treatment.

In contrast in 2015, foliar damage was estimated using the ImageJ image processing software (Java and National Institutes of Health, USA). For this, in each plot one leaf per plant was randomly selected from each of the 8 central plants (total 8 leaves per plot). Leaves were collected at the end of the trial, scanned using a conventional scanner and analyzed using ImageJ software. The percentage of each leaf that had been consumed by *C*. *chalcites* was calculated based on the entire leaf area.

### Statistical analyses

When necessary percentage values for foliar damage were normalized by arcsine transformation. The percentage of efficacy and the percentage of foliar damage increase were subjected to analysis of variance (ANOVA) and mean separation by Tukey’s test (P<0.05), using the Statistix v.10 package (Analytical Software, Tallahassee, FL, USA). Mortality of larvae collected from treated and control plants and reared in the laboratory at sequential times post-application was subjected to repeated measures analysis of variance (ANOVA).

## Results

### Prevalence and diversity of ChchNPV infection in *C*. *chalcites* populations

A total of 4,438 larvae were collected from greenhouse and open-field banana crops, across 30 different sites on the islands (Table 1). The majority of larvae were collected in Tenerife (57%), followed La Palma (18%), Gran Canaria (13%), El Hierro (8%,) and La Gomera (4%). Of these, 103 larvae (2.3%) developed the characteristic signs of lethal polyhedrosis disease during laboratory rearing. None of the larvae were observed with signs of polyhedrosis disease during the process of collecting insects in the field. Overall, 95 (2.1%) larvae died due to parasitism, mostly by larval endoparasitoids such as *Cotesia* spp. or *Hyposoter* spp. Parasitism was ~3-fold higher in larvae collected in open-field than in greenhouse conditions, but was not analyzed in detail.

Of the virus diseased larvae, 56 were collected on Tenerife, 37 on El Hierro, 9 on La Palma, a single larva on La Gomera and no larvae on Gran Canaria (Table 1). The prevalence of infection varied from 0–4.8% on Tenerife and was usually low (0–2%) on the other islands, except for a single site on El Hierro where 13.8% of larvae were infected (37 out of 269 insects collected). The number of sites at which infected larvae were collected varied significantly between islands, from 11 out of 17 sites on Tenerife to a minimum of 0 out of 7 sites on Gran Canaria (Fisher's exact, P = 0.029).

The influence of the greenhouse structure on the prevalence of virus infection was determined by examining the number of infected and healthy larvae collected from each type of production system using the values given in Table 1. Sites at which both open-field and greenhouse structures were sampled were classified as greenhouse samples. The overall prevalence of infection in greenhouse structures was 3% (91 infected out of 3027 larvae sampled), which was twice the prevalence of infection in larvae collected in open-field sites (1.4%, 12 infected out of 846 larvae sampled) (χ^2^ = 6.44, df = 1, P = 0.011, not including sites on Gran Canaria on which no infected larvae were collected). This relationship remained significant even when all mixed sites (open-field and greenhouse) were removed from the analysis (χ^2^ = 6.63, df = 1, P = 0.010).

Of the 103 virus infected larvae, 93 isolates could be identified by their restriction profile. The majority of isolates (N = 76) were identified as ChchNPV-TF1, representing 82% of the identified isolates. ChchNPV-TF2 was the next most prevalent with 11 isolates (12%), followed by ChchNPV-TF3 and ChchNPV-TF5 profiles with 3 isolates each (3% each). The previously characterized ChchNPV-TF4 variant restriction profile was not observed in any of the infected larvae. The ChchNPV-TF1 isolate was present in the islands of Tenerife (37 isolates), El Hierro (33 isolates) and La Palma (6 isolates), while ChchNPV-TF2 was present on Tenerife (10 isolates), and La Palma (1 isolate). ChchNPV-TF3 was present at low prevalence on Tenerife, La Gomera and El Hierro (1 isolate on each island). Finally, ChchNPV-TF5 was only present on El Hierro (3 isolates). Variants ChchNPV-TF1, -TF2, -TF3 and -TF5 were collected from insects in greenhouse production systems, whereas variants -TF1, -TF2 and -TF3 were present in insects from open-field sites. As ChchNPV-TF1 was the most abundant and widespread variant on the archipelago, and was previously showed to be highly infective [5], a single ChchNPV-TF1 isolate from Caldera del Rey in Tenerife was selected for field assays, as the majority of ChchNPV-TF1 isolates were found in Tenerife, especially at the Caldera del Rey site.

### Determining the optimal ChchNPV-TF1 concentration

**Fig 1:**
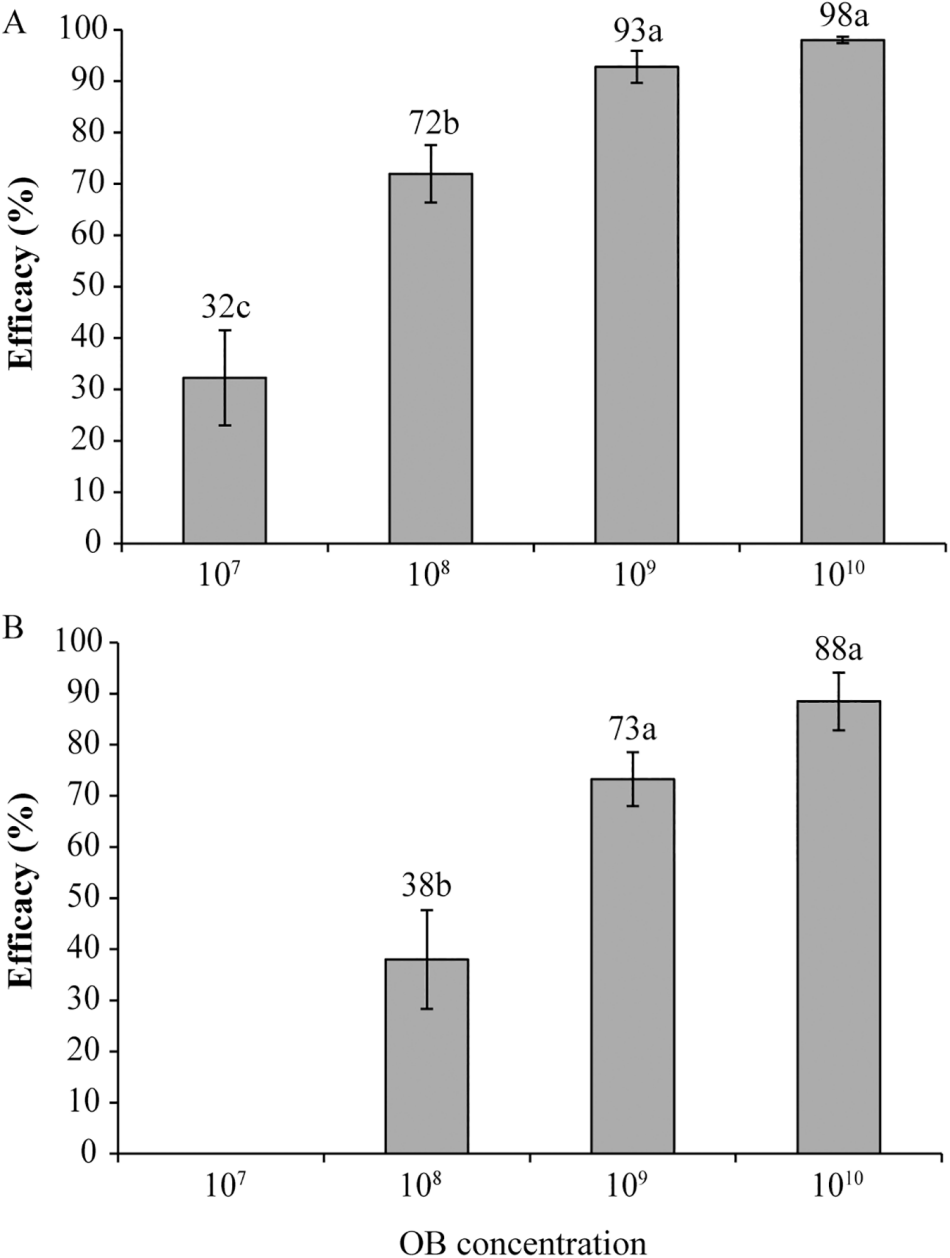
Insecticidal efficacy of ChchNPV-TF1 concentrations. Percentage of insecticidal efficacy of different concentrations of ChchNPV-TF1 in greenhouse (A) and open-field (B) trials. Values followed by identical letters did not differ significantly (ANOVA, Tukey's test, P>0.05). Vertical lines indicate the standard error.

In terms of efficacy, significant differences were observed between the different viral concentrations applied both in greenhouse (F_3,12_ = 40.47, P<0.01) (Fig 1A) and open-field plots (F_2,9_ = 13.19, P = 0.002) (Fig 1B). The two highest concentrations, 10^9^ and 10^10^ OBs/l, were similarly effective at controlling *C*. *chalcites* in both types of setting (Tukey's test, P>0.05), with up to 98% and 88% efficacy under greenhouse and open-field conditions, respectively. Application of 10^8^ OB/l resulted in intermediate control efficacy (38–72% depending on setting), whereas 10^7^ OB/l resulted in zero control efficacy of *C*. *chalcites* in the open-field and 32% control efficacy in greenhouse grown plants (Fig 1A and 1B).

**Fig 2:**
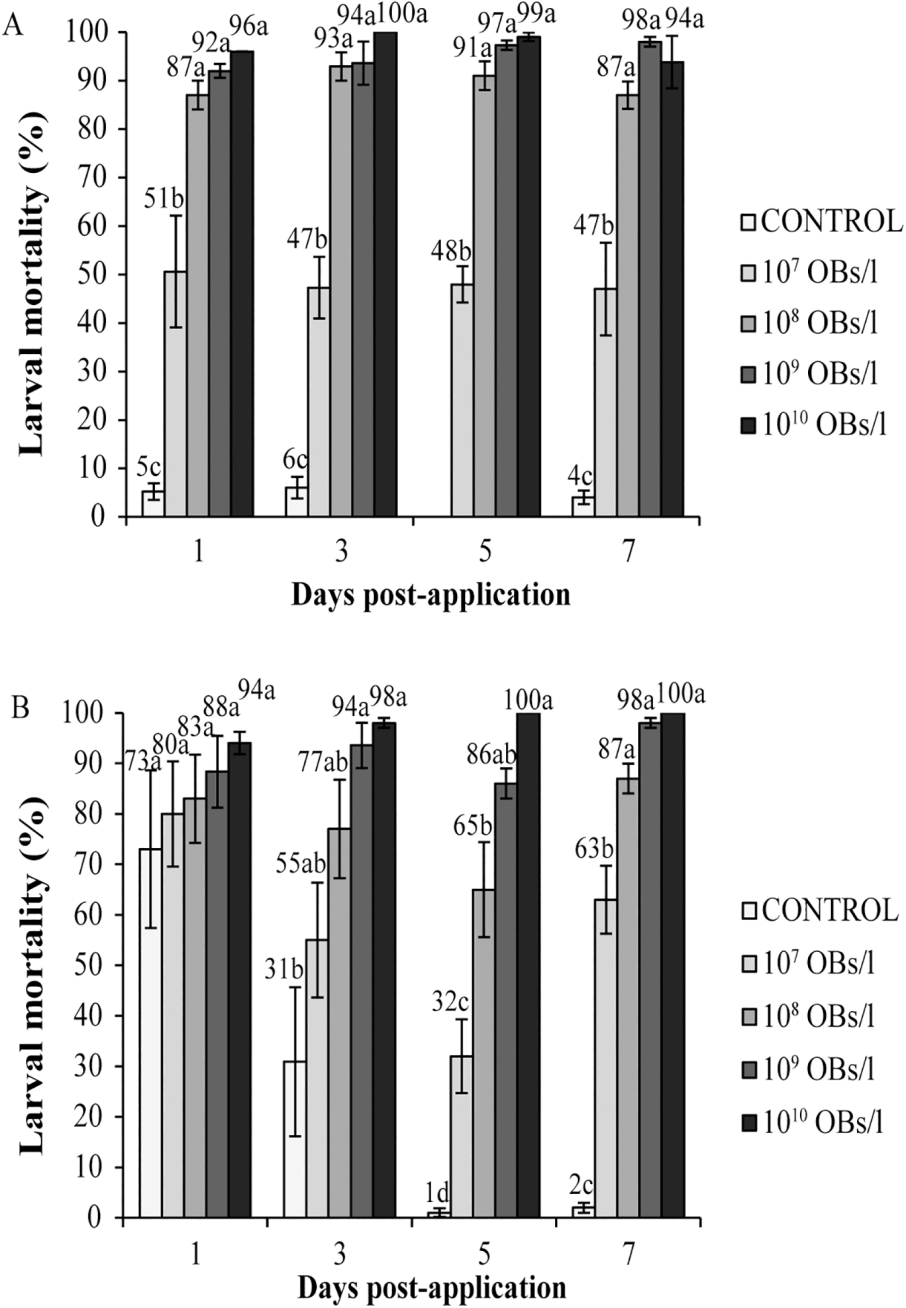
Percentage of larval mortality over time. Percentage of larval mortality in insects collected at different times after treatment with different concentrations of ChchNPV-TF1 in greenhouse (A) and open-field (B) trials. Insects were reared in the laboratory until death or pupation. Values followed by identical letters did not differ significantly for comparisons of treatments within each time point (repeated-measures ANOVA, Tukey's test, P>0.05). Vertical lines indicate the standard error.

In relation to the larval mortality produced at different time points after treatments, the different OB concentrations produced variable mortalities across the different time points both in greenhouse (Fig 2A) and open-field plots (Fig 2B). In the greenhouse trial, the three highest viral concentrations, 10^8^, 10^9^ and 10^10^ OBs/l, produced similar mortalities at different time points, between 87% to 100% (Tukey´s test, P>0.05), which were significantly higher than mortalities observed at the lowest concentration 10^7^ OBs/l (Tukey´s test, P<0.05) which fluctuated around 50% during the 7 days of the trial. In contrast, in the open-field, the two highest concentrations produced similar mortalities of 86–100% (Tukey's test, P>0.05), whereas mortality in the lowest concentration treatment (10^7^ OB/l) declined rapidly following a peak of 73% at day 1, and the 10^8^ OB/l treatment fluctuated between 80 and 31% mortality during the trial (Fig 2B). DNA extracted from each group of dead larvae showed the same profile as the ChchNPV-TF1 variant, confirming that larvae died due to ChchNPV-TF1 infection.

According to these results, the concentration of 10^9^ OBs/l was selected as the most suitable concentration for subsequent trials in greenhouses and open-field plots, since this concentration produced mortalities similar to those of the highest concentration tested.

### Insecticidal efficacy of ChchNPV-TF1 in greenhouse and open-field trials

**Fig 3:**
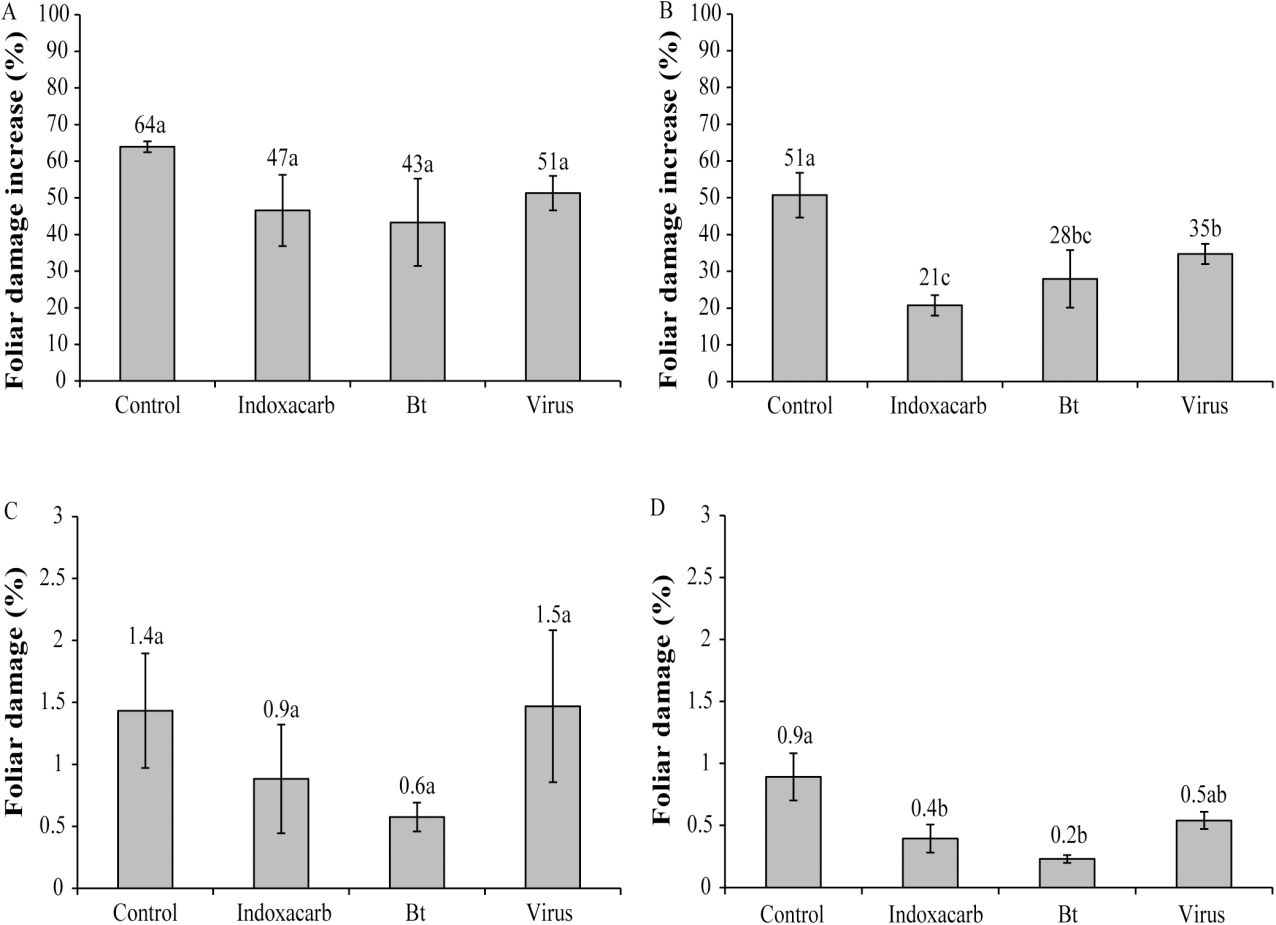
Foliar damage. Percentage increase in foliar damage produced by *C*. *chalcites* in greenhouse (A) and open-field (B) in 2013 trials, and percentage of foliar damage in greenhouse (C) and open-field (D) trials in 2015. Values followed by identical letters did not differ significantly (ANOVA, Tukey's test, P>0.05) for comparisons of treatments. Vertical lines indicate the standard error.

Under greenhouse conditions the percentage increase in foliar damage did not differ significantly between the different treatments and control plots, with a 43–64% increase in foliar damage across all treatments and control in 2013 (F_3,66_ = 2.37, P = 0.08) (Fig 3A), In open-field trials in 2013, the increase in foliar damage varied significantly among treatments (F_3,124_ = 17.64, P<0.01) (Fig 3B). Specifically, foliar damage increased significantly less in the ChchNPV-TF1 treatment and the Bt treatment compared to the control (Tukey's test, P<0.05), whereas the indoxacarb treatment resulted in the lowest increase in foliar damage in 2013 (Tukey's test, P<0.05) (Fig 3B).

In trials performed in 2015 percentage of foliar damage was estimated (rather than the increase in damage that was measured in the previous 2013 trials). In greenhouse trials performed in 2015 percentage of foliar damage ranged from 0.6–1.5% and did not differ significantly among treatments (F_3,44_ = 1.44, P = 0.24) (Fig 3C). In contrast, in open field trials in 2015 foliar damage was significantly lower in treated plots than control plots (F_3,60_ = 5.78, P = 0.001) (Fig 3D), with the lowest foliar damage in the indoxacarb and Bt treatments and intermediate damage in the ChchNPV-TF1 treatment (Tukey's test, P>0.05) (Fig 3D).

**Fig 4:**
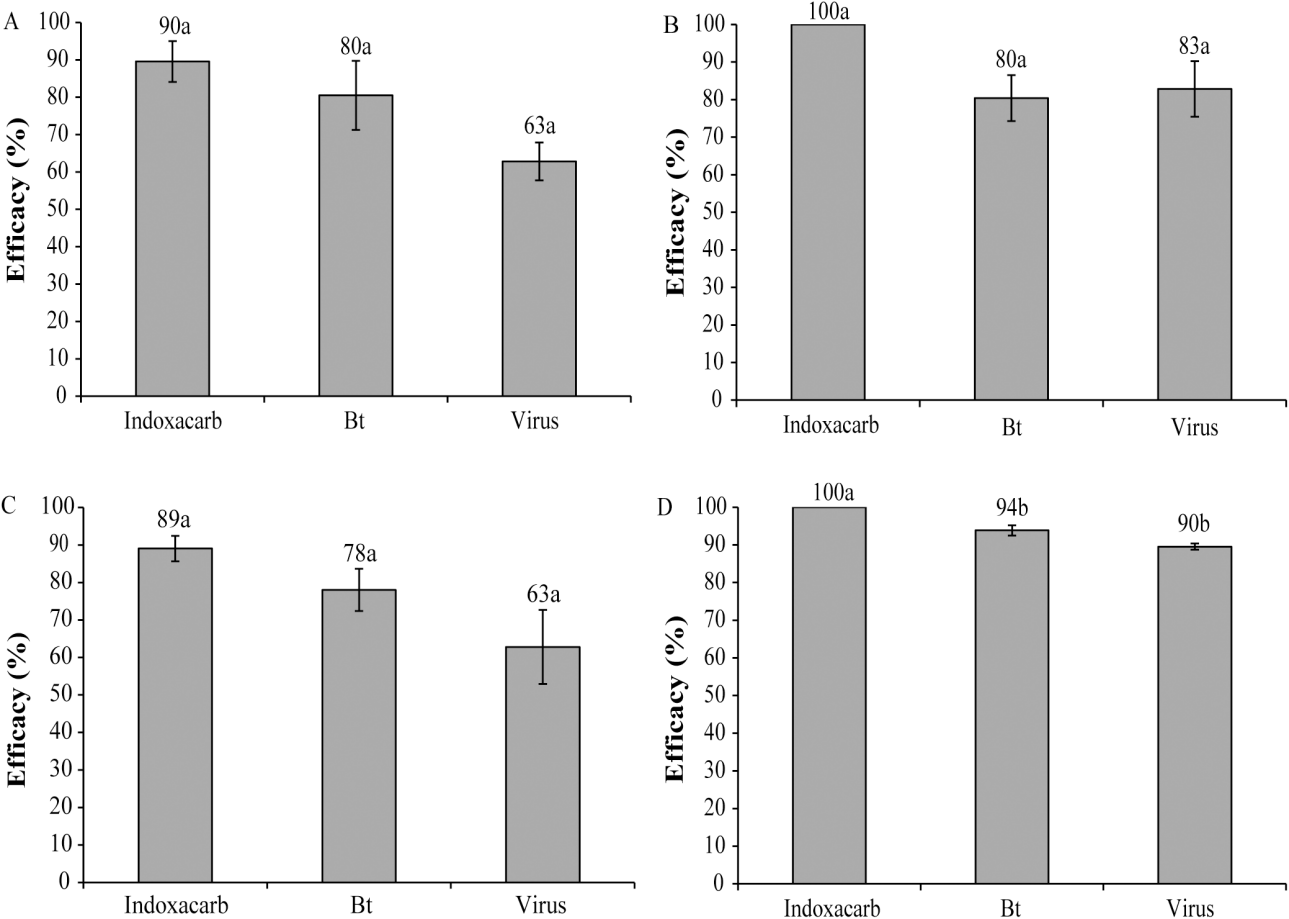
Insecticidal efficacy. Percentage of insecticidal efficacy of different treatments in greenhouse (A) and open-field (B) trials in 2013 and in open-field (C) and greenhouse (D) trials in 2015. Values followed by identical letters did not differ significantly (ANOVA, Tukey's test, P>0.05). Vertical lines indicate the standard error.

The insecticidal efficacy of ChchNPV-TF1 OBs in 2013 was similar to that of the other insecticides used in the greenhouse trial (F_2,6_ = 2.63, P = 0.15) (Fig 4A) and open-field trial (F_2,9_ = 3.67, P = 0.07) (Fig 4B). Similarly, in 2015 ChchNPV-TF1 was as effective as Bt and indoxacarb in open-field plots (F_2,9_ = 2.79, P = 0.11) (Fig 4C), whereas in greenhouses the ChchNPV-TF1 treatment was slightly less effective than indoxacarb but similar to that of Bt (F_2,6_ = 21.33, P = 0.002) (Fig 4D).

**Fig 5:**
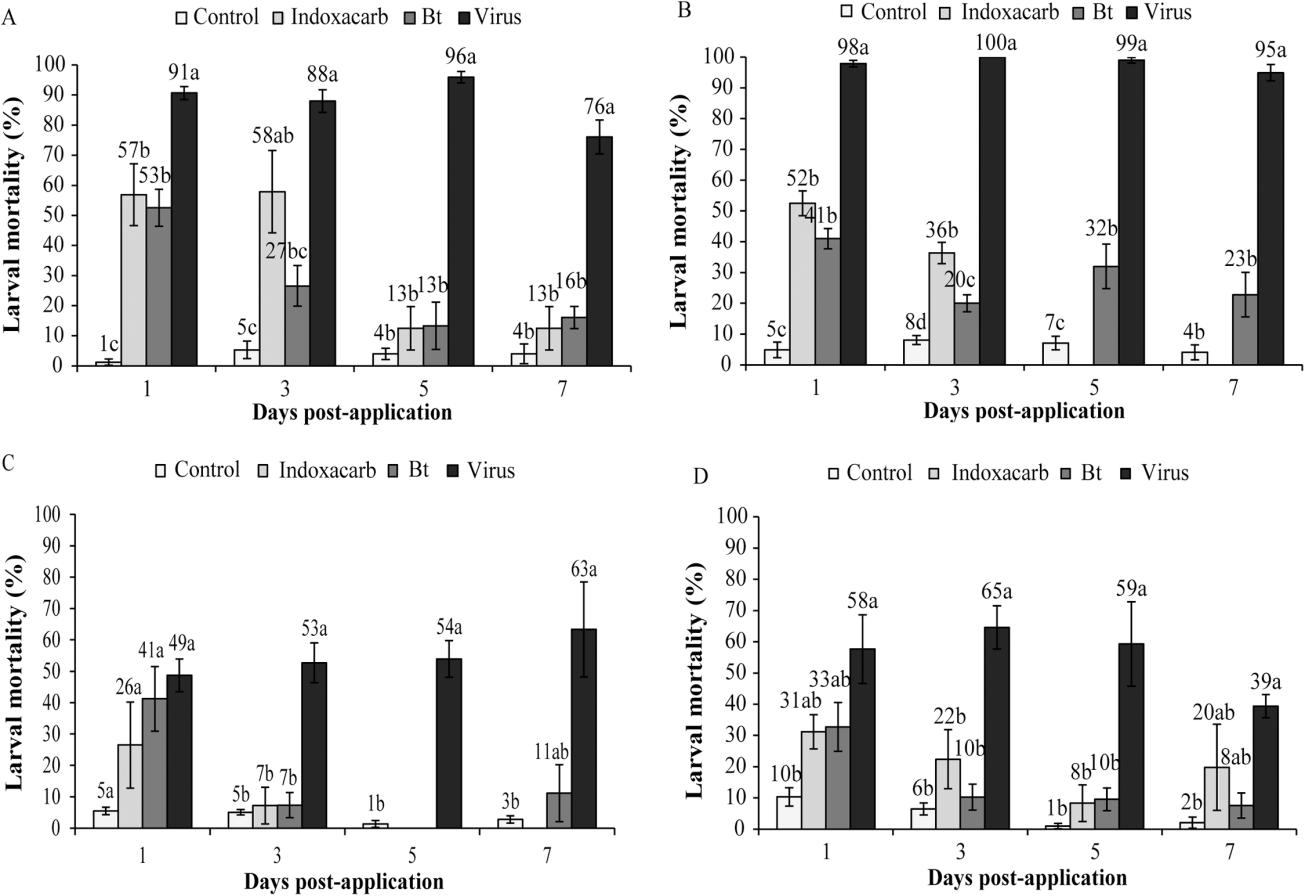
Percentage of larval mortality over time. Percentage of larval mortality in laboratory-reared larvae collected at different time intervals after treatments in greenhouse (A) and open-field (B) trials in 2013 and in greenhouse (C) and open-field (D) trials in 2015. Values followed by identical letters did not differ significantly for comparisons of treatments within each time point (repeated-measures ANOVA, Tukey's test, P>0.05). Vertical lines indicate the standard error.

ChchNPV-TF1 was the only treatment that resulted in a high prevalence of mortality in larvae collected over time. In 2013, control mortality was consistently low (1–8%) in insects collected over time from both greenhouse (Fig 5A) and open-field plots (Fig 5B). Insects from ChchNPV-TF1 treated plots experienced 76–96% mortality during laboratory rearing in samples from the greenhouse trial (Fig 5A) and 95–100% in the open-field trial (Fig 5B). In contrast, insects from the indoxacarb and Bt treated plots had an intermediate prevalence of mortality during laboratory rearing, which tended to decrease over time, from 53–57% (day 1) to 13–16% (day 7) in larvae collected from the greenhouse plots (Fig 5A) and 41–52% (day 1) to 0–23% (day 7) in the open-field plots (Fig 5B). The low mortality observed in larvae collected in indoxacarb and Bt treatment was likely due to the rapid action of these insecticides in comparison with the virus, resulting in an initial peak of mortality that declined over time.

During the trials performed in 2015, insects from the control treatment experienced low mortality during laboratory rearing in the greenhouse (1–5%) and open-field trial (1–10%) (Fig 5C and 5D). Mortality during laboratory rearing varied in the ChchNPV treatment in the greenhouse trial (49–63% mortality) and the open-field trial (39–65% mortality), which was generally higher than mortalities observed in the other insecticide treatments. Laboratory-reared larvae from both indoxacarb and Bt treatments initially experienced intermediate prevalence of mortality in the greenhouse (26–41%) and open-field trail (31–33%), but mortality declined in samples taken during the trails both in the greenhouse (Fig 5C) and open-field plots (Fig 5D). At some time points near to the end of the trials (at day 5 and 7 post-application), no larvae were present on experimental plants treated with conventional insecticides, likely due to the insecticidal activity and rapid action of Bt (Fig 5C) and indoxacarb (Fig 5B and 5C).

## Discussion

The present study aimed to evaluate the natural diversity of ChchNPV on the Canary Islands and the efficacy of this virus to control *C*. *chalcites* populations on young banana plants both in greenhouses and open-field trials. Overall, 2.3% of larvae collected from natural infestations of *C*. *chalcites* on banana plants died from ChchNPV infection during laboratory rearing, although cadavers with the characteristic signs of polyhedrosis disease were never observed in the field (except during field trials following application of the virus). In a smaller scale study in 2006 involving surveys of *C*. *chalcites* larvae on the islands of Tenerife, La Palma, Gran Canaria and El Hierro, overall 2.5% died due to lethal polyhedrosis disease [5]. This rather low prevalence of enzootic infection is similar to that reported in other nucleopolyhedrovirus pathosystems such as the nucleopolyhedrovirus of *Spodoptera frugiperda* (SfMNPV) on maize in Mexico [17], Colombia [18] or Brazil [19]. In contrast, high prevalence of infection and, on occasions, epizootics of lethal disease, have been detected in other nucleopolyhedrovirus-insect pathosystems, particularly in high density insect populations [20–22].

Parasitism in laboratory-reared larvae was 2.1% overall, indicating a modest contribution of larval parasitoids to the control of *C*. *chalcites* populations at densities at which growers are likely to apply insecticidal control measures. Very similar results were obtained during a small scale survey performed in 2006 in which 2.3% of field-collected larvae died from parasitism [5].

The majority of virus-infected *C*. *chalcites* larvae were collected on Tenerife (54%) and El Hierro (36%), in agreement with a previous study in which 69% and 22% of infected larvae were collected on Tenerife and El Hierro, respectively [5]. Although the highest numbers of larvae were collected on Tenerife (indicating a higher prevalence of infestation of banana crops by this pest on this island), there was no clear relationship between numbers of larvae collected and the prevalence of virus infection detected during laboratory rearing.

In contrast to parasitism, which was more prevalent in open-field collected larvae than in greenhouses (3:1), the prevalence of virus infection in greenhouse structures was twice that in open-field sites. This may in part be due to higher densities of infestation under greenhouse conditions, given that four-fold more larvae were collected during greenhouse compared to open-field sampling. However, a more likely explication resides in the protection from ultraviolet light provided by the greenhouse structure. Baculoviruses are rapidly inactivated by ultraviolet light [23] and plastic greenhouses effectively filter a large part of the ultraviolet spectrum [24, 25]. Although the effect of the mesh-built greenhouses used for banana production on incident ultraviolet light was not measured in the present study, it seems likely that reduced ultraviolet radiation on banana crops under plastic mesh could favor the conservation of ChchNPV OBs on foliage and in the upper layers of soil, resulting in a higher prevalence of virus infection in larvae compared to those feeding in open-field plantations.

In line with our previous study [5] ChchNPV-TF1 was the most prevalent variant in the Canary Islands. The ChchNPV-TF2, ChchNPV-TF3 and ChchNPV-TF5 variants were isolated from larvae collected across the different islands, whereas previously we had only found these variants on southern Tenerife [5]. The prevalence of the ChchNPV-TF1 variant over that of other variants and its presence at numerous sites across the islands, in addition to its high pathogenicity [5], led us to use this variant in field trials. Previously we developed a specific mixture of ChchNPV genotypes with increased pathogenicity and virulence that has been the subject of a European Patent [26]. The stability of the specific genotype mixture was corroborated in the laboratory but not in field conditions, which may favour transmission of different proportions of certain genotypes resulting in changes to the overall phenotypic characteristics [13, 21, 22, 27]. Clearly this issue requires empirical testing but the ChchNPV-TF1 variant provided a useful model against which we will be able to evaluate the insecticidal efficacy of the specific genotype mixture in the future.

The insecticidal efficacy of the ChchNPV-TF1 variant was compared in greenhouse and open-field conditions, as ~65% of the banana production is produced in open-field conditions in the Canary Islands [1], and we had not previously performed such a comparison [10]. As greenhouse conditions tend to be more stable than those of open-field crops [28, 29], the harsher conditions of open-field crops might have implications for the quantities of viral OBs required for effective control or may affect the persistence of viral OB treatments on crop foliage [30]. Contrary to our expectations, application of 10^9^ OBs/l resulted in high mortality of *C*. *chalcites* larvae both in greenhouse and open-field banana plants. Previous studies had indicated that 10^9^ OBs/l might be sufficient to protect banana plants from *C*. *chalcites* feeding damage under greenhouse conditions [10]. This concentration of viral OBs is similar to that reported in other baculoviruses used as biological insecticides, such as those of nucleopolyhedroviruses applied for the control of *Spodoptera* spp. in greenhouse and field crops [30–32]. As the leaf area and presence of fruit in the crop changes during growth the volume required to treat large plants tends to increase so that the overall quantity of OBs applied per hectare depends on the growth stage and crop phenology. For example, in the present study one liter of spray suspension at 10^9^ OBs/l was used to treat 25 m^2^ of young banana plants. As adult plants require volumes of up to 2,000 l/ha in commercial plantations [E. Fuentes, pers. communication], this would involve up to 2x10^12^ OBs/ha. This amount is within the range of OB applications typically used for commercial biological insecticides targeted at lepidopteran pests [30].

The high natural larval mortality observed in greenhouse and open-field trials did not mask the efficacy of ChchNPV-TF1 as a biological insecticide. This natural mortality might be due to environmental factors such as wind or high temperatures recorded in greenhouses during the trials (up to 40°C), as well as to the presence of predators or the movement of larvae from experimental plants in search of additional food resources [33, 34]. Generally, ChchNPV-TF1 was as effective as conventional treatments reducing foliar damage and number of larvae under greenhouse and open-field conditions. We had previously observed that application of 10^8^ OB/l of the ChchNPV-TF1 variant was as effective as indoxacarb and Bt treatments in controlling pest infestation and foliar damage under greenhouse conditions [10]. Indeed, applications of 10^9^ OBs/l were significantly more effective than the conventional treatments in the previous study. The reasons for this higher efficacy remain unclear. This may be due to variation in environmental conditions as previous field trials were performed in autumn, whereas those of the present study were performed during summer months during periods of high temperatures and strong sunlight that could have reduced OB persistence on plants. The insect colony used to infest experimental plants in the present study may also have been less susceptible to infection than colony used during the previous study, as strain variation in insect susceptibility to pathogens is a well-recognized phenomenon [35].

Nonetheless in the present study, the insecticidal efficacy of ChchNPV was clearly demonstrated under both greenhouse and open-field conditions. Indeed, under certain conditions baculoviruses can be as effective as chemical insecticides, although other characteristics such as their specificity, persistence in insect populations and their ability to control pests that are resistant to chemical insecticides make them uniquely valuable pest control agents in a range of situations [9, 36, 37]. However, the efficacy of the virus in protecting banana fruit from *C*. *chalcites* feeding damage remains to be tested. This is crucial because foliar feeding by this pest has little effect on banana yields whereas direct damage to bananas totally eliminates the commercial value of scarred fruit. Experiments are in progress to evaluate the efficacy of the virus in plantations of fruiting adult plants.

Application of ChchNPV-TF1 OBs resulted in an extended period of larval mortality in larvae collected and reared in the laboratory. Most larvae died 5–7 days after application of the virus, but the fact that larvae collected at up to 7 days post-treatment continued to show high levels of lethal virus disease during laboratory rearing suggests that they had acquired a lethal dose of the virus several days after application of the virus. In contrast, indoxacarb and Bt treatments resulted in a rapid peak in mortality that declined over the 7-day sampling period, reflecting the different modes of action of these products compared to that of the virus. Similar results have been observed in samples taken over time in crops treated with baculoviruses and conventional insecticides for control of *Spodoptera exigua* in greenhouses [25, 38, 39] or *Helicoverpa armigera* [40, 41]. These results indicate a greater persistence of ChchNPV-TF1 OBs on the banana plant with respect to conventional treatments. Therefore, ChchNPV may provide an extended period of pest control, producing larval mortality for longer, compared to the other insecticides used in our study.

Currently, a low number of active substances are authorized for *C*. *chalcites* control in banana crops, with indoxacarb and Bt var. kurstaki being the most frequently used products [E. Fuentes, pers. communication]. ChchNPV-TF1 provides an attractive alternative to Bt for *C*. *chalcites* control as a highly effective and highly specific insecticide that does not leave xenobiotic residues in fruit and is compatible with IPM systems that aim to conserve natural enemy populations [8, 34, 42]. ChchNPV-TF1 based products could be incorporated into integrated pest management programs, given the compatibility of this virus with biological and chemical control measures, thus reducing farmer dependence on synthetic insecticides and thereby reducing the likelihood of the development of insecticide resistance in the pest population. As a low number of substances are authorized and those are used repeatedly, the market for a ChchNPV-TF1-based product is well defined and could be commercially viable if adopted by banana growers on the islands.

## Supporting information

S1 File Fuentes EG Letter. A letter written by the relevant author, Fuentes EG, which supported personal communications.
(PDF)

S2 File Original data. An excel file that included all the original data from all the experiments.
(XLSX)
