# Supplementary material for: Chrysodeixis chalcites nucleopolyhedrovirus (ChchNPV): Natural occurrence and efficacy as a biological insecticide on young banana plants in greenhouse and open-field conditions on the Canary Islands
Source: PLoS One. 2017 Jul 27;12(7):e0181384. doi: 10.1371/journal.pone.0181384 (PMC5531463; doi:10.1371/journal.pone.0181384)

9 May 2017

To whom it may concern:

The manuscript submitted to PLoS ONE is the second manuscript of my PhD. The first described the incidence, damage and costs of *Chrysodeixis chalcites* control in banana crops in the Canary Islands. My PhD has focused on determining several aspects relevant for the implementation of integrated pest management against *C. chalcites* in banana crops. Therefore, logically this second manuscript includes some information that was obtained and is now under consideration for publication elsewhere.

The first MS describes a high prevalence of *C. chalcites* infestations (42-100%) on the Canary Islands. I also measured mean foliar and fruit damage, which varied significantly across islands, plantation aspect and season. I estimated the weight of *C. chalcites* damaged bananas (0.2 to 4.2%), representing an overall loss of 3,155 tonnes bananas/yr due to *C. chalcites* damage, representing 1.5% of annual production or 2.68 million €/yr on the islands. Finally, the treatment volume and cost of *C. chalcites* control were also measured. The most frequently used pesticides were indoxacarb and *Bacillus thuringiensis* var. kurstaki, usually applied on an average of 2,000 liter/ha on three occasions per crop cycle, at a cost of approximately 240 €/ha.

According to the results of this first study, I found that the direct damage that *C. chalcites* causes to banana fruit results in significant economic losses and that effective and sustainable control strategies are required against this pest. Therefore, this second study focused on determining the efficacy of the baculovirus (ChchNPV) in control of *C. chalcites*, with the aim in incorporating this biological control agent in integrated pest management programs.

Sincerely,

Ernesto G. Fuentes

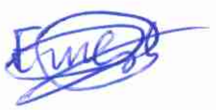

Supplement: S1 File — A letter written by the relevant author, Fuentes EG, which supported personal communications. (PDF) [file pone.0181384.s001.pdf]
